# Patterns of Dietary Iron Intake, Iron Status, and Predictors of Haemoglobin Levels among Early Adolescents in a Rural Ghanaian District

**DOI:** 10.1155/2020/3183281

**Published:** 2020-12-24

**Authors:** Michael Akenteng Wiafe, Charles Apprey, Reginald Adjetey Annan

**Affiliations:** Department of Biochemistry and Biotechnology, Kwame Nkrumah University of Science and Technology, Private Mail Bag, Kumasi, Ghana

## Abstract

**Introduction:**

Early adolescents are vulnerable to anaemia due to lean body mass and menarche. The study assessed patterns of dietary iron intake, iron status, and predictors of anaemia among early adolescents.

**Method:**

One hundred and thirty-seven early adolescents were randomly selected in a rural district in Ghana. Multiple-pass 24-hour recall, iron food frequency questionnaire consisting of 27 food items, and semistructured questionnaire were administered. Variables include sociodemographics, dietary factors, and laboratory investigation including haemoglobin, ferritin, and C-reactive protein examination. Statistical Package for the Social Sciences (SPSS) software was used to calculate odds ratio and perform Mann–Whitney U test, chi-square (X^2^) test, exploratory factor analysis, and partial correlation (*r*) tests.

**Results:**

Participants had mean age of 11.5 years. Three iron dietary patterns explaining 28.7% of the total variance were identified: iron dietary pattern 1 (11%) composed of iron-rich, iron-enhancing, and iron-inhibiting foods; iron dietary pattern 2 (9.9%) comprised of iron-rich, iron-enhancing, and non-iron-inhibiting foods; and iron dietary pattern 3 (7.1%) consisting of stinging nettle, iron-inhibiting foods, non-iron-enhancing foods, non-cocoyam leaves, and non-turkey berries. Meal skipping (*X*^2^ = 5.7, *p* < 0.05), times of eating a day (*X*^2^ = 12.6, *p* < 0.05), and guardian educational status (*X*^2^ = 6.7, *p* < 0.05) significantly affected dietary iron intake. Anaemia was associated with meal skipping (*β* = 0.367, *p* > 0.05), snacking (*β* = 0.484, *p* > 0.05), and junior high school (JHS) education (*β* = 0.544, *p* > 0.05). Partial correlation showed statistically significant relationship between iron dietary pattern 1 and dietary iron (*r* = −0.234, *p* < 0.01), iron dietary pattern 2 and dietary iron (*r* = -0.198, *p* < 0.05), iron dietary pattern 2 and vitamin C (*r* = -0.201, *p* < 0.05), and haemoglobin and ferritin (*r* = −0.178, *p* < 0.05).

**Conclusion:**

Meal skipping, guardian educational status, and number of times of eating a day were significantly associated with dietary iron intake. Meal skipping, snacking, and adolescents with JHS education were positively associated with anaemia.

## 1. Introduction

World Health Organization (WHO) defines adolescence as a period between 10 to 19 years of the life cycle, categorised into early, middle, and late adolescence, with about three-quarters living in developing countries [1, 2]. Early adolescents, particularly females, are among those at high risk of iron deficiency due to menarche, growth spurt, and other physiological development [3–6]. A study has recommended the consideration of iron deficiency anaemia as one of the key nutritional status indicators in adolescence [7]. Iron is relevant for immunity, cognitive function, behaviour, physical activity, and lean body mass development [8–12].

Dietary iron is obtained from a restricted number of animal and plant sources of food, mostly from meat, liver, legumes, and nuts [13]. Non-haem iron sources such as cereals and grains products were the main dietary iron intake of the studied population in Spain [14]. In developing countries, plant-based food forms a major part of the diet with limited quantities from animal sources, and increased consumption of western diet worsens the adolescent micronutrient plight [15, 16]. Insufficient dietary iron intake contributed inversely to iron status indicators such as serum ferritin, transferrin saturation, and total iron-binding capacity [17].

The prevalence of anaemia among school age children (5–14 years) is estimated to be 25.4% globally [18]. In least developed countries, about 27% of adolescents suffered from anaemia related to iron deficiency [19]. In Nepal, a nationwide study on anaemia among adolescents showed that 38% of females and 24% of males had iron deficiency anaemia [20]. In the literature, a study showed that 24% of adolescent girls (10–19 years) and 13% of adolescent boys were anaemic in Ghana [21]. The data shows that prevalence of anaemia in adolescents is a public health concern. Much research has not been done about the prevalence of anaemia and its contributory factors among early adolescents in Ghana.

Adolescent health is influenced by prenatal and early childhood development [22]. Abuse of alcohol, sexual promiscuity, attention disorders, and lawlessness in adolescence have been linked to childhood anaemia [23]. Adolescents having iron deficiency with or without anaemia showed low scholastic, verbal learning, attention, mental balance, and recognition performance [24]. Low birth weight and low Apgar score have been the pregnancy outcomes of pregnant adolescents with abnormal iron status [25].

Low levels of guardian education, low socioeconomic status, being female adolescent, early age of menarche, prolonged and excessive blood loss during menstruation, low intake of iron-fortified foods, inadequate intake of haem foods and iron-enhancers, and high intake of iron-inhibitors are the drivers of iron deficiency anaemia in adolescents [26–32]. Poor dietary exploration, worm infestations, meal skipping, illiteracy, seeking peer approval, and concern for body shape predispose adolescents to iron malnutrition particularly those living in least developed countries [33–36]. The present study aimed to identify patterns of dietary iron intake, iron status, and predictors of anaemia among early adolescents in a rural district in Ghana.

## 2. Materials and Methods

### 2.1. Study Design

A cross-sectional study was conducted at the Asante Akim South Municipality. The study protocol was approved by the Committee on Human Research Publication and Ethics at Kwame Nkrumah University of Science and Technology, Kumasi, and permission was also given by the Asante Akim South Municipal Health Directorate.

### 2.2. Participants

Early adolescents (10–14 years) were approached in the municipality for the study. One hundred and thirty-seven participants gave their assent and guardians gave their consent after the objective of the study was explained in the language they understand. Participants and guardians signed an informed consent form. Those who could not sign were made to use thumbprint.

### 2.3. Data Collection

One hundred and thirty-seven participants responded to the dietary questionnaire. One participant failed to return to the phlebotomy site for blood sample collection but responded to the questionnaire. Five participants had their haemoglobin checked: one had inadequate serum for the analysis of ferritin and C-reactive protein; four participants' samples were double labelled and therefore removed from the analysis. Thus, a total of 136 participants had their haemoglobin checked, and 131 had their ferritin and C-reactive protein analysed.

### 2.4. Dietary Survey

Nutritionists and dieticians were trained to use 24-hour recall to collect dietary data, on one weekend and two weekdays. Participants were shown food models to estimate how much they consumed, and results were documented. All the foods eaten were converted into grams, and total dietary iron intake was analysed using the nutrient analysis template of foods in Ghana. Adequate dietary iron was defined as 8 mg/day as recommended by the National Academy of Sciences [37].

### 2.5. Dietary Patterns

Iron Food Frequency Questionnaire contained 27 food items: 14 iron-rich foods; 8 iron-enhancing foods; 5 iron-inhibiting foods. The questionnaire had the following options for participants to choose from: a. daily; b. weekly; c. monthly; d. occasionally/never.

### 2.6. Blood Analysis

Venous blood samples of participants were analysed for ferritin, C-reactive protein, and haemoglobin (Hb). Human ferritin Elisa test kit (Enzyme Immunoassay test kit, catalogue number: 10601) was used for the ferritin analysis, and high sensitivity C-reactive protein (Enzyme Immunoassay test kit, catalogue number: 10603) was used for C-reactive protein at the Clinical Analysis Laboratory at the Kwame Nkrumah University of Science and Technology, Kumasi.

#### 2.6.1. Procedure for Human Ferritin Analysis

Twenty (20) microlitres (*μ*L) of samples, standard specimens, and controls were appropriately dispensed into wells. One hundred (100) microlitres (*μ*L) of enzyme conjugate reagent was dispensed into each well. The content in the wells were thoroughly mixed for thirty (30) seconds. The wells were incubated at room temperature (18–22°C) for sixty (60) minutes. The incubated mixture was removed by flicking plate content in a waste container. The microlitre wells were rinsed and flicked five (5) times with washing buffer (1X). The wells were stroked sharply onto absorbent paper to remove all residual water droplets. 100 *μ*L of tetramethylbenzidine (TMB) substrate was dispensed into each well and was gently mixed for five (5) seconds. The content was incubated at room temperature in the dark for twenty (20) minutes. The reaction was stopped by adding 100 *μ*L of stop solution to each well. Each was gently mixed for thirty (30) seconds, and it was ensured that all the blue colour changed to yellow colour completely. The optical density was read at 450 nm with a microlitre reader within fifteen minutes.

#### 2.6.2. Procedure for C-Reactive Protein Analysis

Participants serums and control serums were diluted with distilled water (100-fold) prior to use. Fifty (50) *µ*L of undiluted CRP standards, diluted specimens, and diluted controls were dispensed into appropriate wells. 50 *µ*L of sample diluent was dispensed into each well and thoroughly mixed for 30 seconds. It was ensured that the content was mixed completely. The mixture was incubated at room temperature (18–22°C) for thirty (30) minutes. The incubated mixture was removed by flicking plate contents into a waste container. The microlitre wells were rinsed and flicked 5 times with washing buffer (1X). The wells were stroked sharply onto absorbent paper to remove all residual water droplets. One hundred (100) *µ*L CRP enzyme conjugate reagent was dispensed into each well. The content was thoroughly mixed for 30 seconds and incubated at room temperature (18–22°C) for 30 minutes. The incubated mixture was removed by flicking plate contents into a waste container. The microtiter wells were rinsed and flicked five times with washing buffer (1X). The wells were stroked sharply onto absorbent paper or paper towels to remove all residual water droplets. 100 µL tetramethylbenzidine (TMB) reagent was dispensed into each well and gently mixed for five (5) seconds. The mixture was incubated at room temperature for 30 minutes. 100 µL of stop solution was added to each well to stop the reaction. The content was gently mixed for 30 seconds to ensure that all the blue colour changed to yellow colour completely. A microtiter well reader at absorbance of 450 nm was used to read the results within fifteen (15) minutes.

#### 2.6.3. Procedure for Haemoglobin Testing

Haemoglobin (Hb) was determined using HemoCue Hb 301 analyser (HemoCue AB, SE-262 23, Ӓngelholm, Sweden) on the field. The microcuvette (301) was filled automatically after placing it in a drop of blood. Lint-free wipe was used to wipe off excess blood on the microcuvette. The open end of the microcuvette was not touched and also checked for air bubbles. The filled microcuvette was placed in the holder, and the cuvette holder was gently touched to automatically slide it to the measuring position. The Hb result was shown within about ten (10) seconds, and it was documented. Anaemia was defined as Hb less than 11 g/dL as given by the manufacturer's manual.

### 2.7. Statistical Analysis

Statistical Package for the Social Sciences (SPSS) (IBM Corp. Released 2017. IBM SPSS Statistics for Windows, Version 25.0. Armonk, NY: IBM Corp) was used for all the statistical analyses. Descriptive statistics were used to analyse sociodemographic characteristics and dietary factors presented in frequency, percentages, and charts. Continuous nonparametric data was presented in the median (minimum and maximum) and independent samples. Mann–Whitney U test was used to analyse haemoglobin and ferritin status. Chi-square test was done to assess the relationship between the dietary iron intake, sociodemographic factors, and dietary factors. Exploratory factor analysis with principal component was used to obtain the iron dietary patterns with eleven components extracted. Three dietary patterns were retained based on eigenvalues greater than one, loading factor of ±0.3, and scree plot. Being adjusted for age and gender, the partial correlation was employed to assess the relationship and direction of iron dietary pattern and biochemical markers. Binary logistics regression was used to predict the relationship between sociodemographic factors, such as gender, level of education, and guardian education status, and dietary factors, such as meal skipping, breakfast, snacking, dietary iron and vitamin C intake, and haemoglobin status. The sociodemographic and dietary factors were the independent variables, and haemoglobin, ferritin, and C-reactive protein were the dependent variables.

## 3. Results

### 3.1. Descriptive Statistics

A total of 137 early adolescents (10–14 years) voluntarily participated in the study, with 50.4% being males and 49.6% females. The majority of the respondents were aged eleven (30.7%), followed by thirteen (24.1%), twelve (23.3%), and ten (21.9%) years. Participants had mean age of 11.5 ± 1.1 years. Among the participants, 70.1% were in primary school and 29.9% were in junior high school (JHS); 77.9% and 22.1% of guardians had formal and nonformal education, respectively.

### 3.2. Meal Patterns of Study Participants

**Figure 1:**
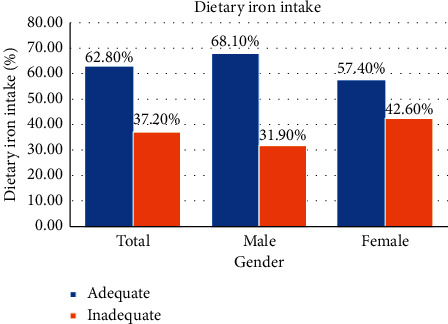
Dietary iron status by gender.

More males (68.1%) had adequate dietary iron intake than females (57.4%) (Figure 1). A higher number of participants whose guardians had formal education (70.9%) had adequate intake of dietary iron than those without formal education (29.1%). Most of the participants ate three times daily (71.5%), followed by four or more times (14.6%) and twice (13.9%). In descending order of adequate dietary iron intake, 68.6%, 22.1%, and 9.3% ate three times, four or more times, and twice daily, respectively. Meal skipping was popular representing 55.5% as against non-meal skipping 45.5%. Most of the non-meal skippers (52.3%) had adequate dietary iron intake compared with meal skippers (47.7%). Lunch (75%) was the most skipped meal by participants, followed by breakfast (19.7%) and dinner (5.3%). More of the lunch skippers (73.2%) had adequate dietary iron intake than the other meals skippers. Among the adolescents who took breakfast, the majority (86%) had adequate dietary iron intake as against inadequate intake (82.4%). Seventy-three percent (73%) of participants snacked daily and 27% did not. The majority of snackers had adequate intake of dietary iron compared with non-snackers (72.1% versus 27.9%) (Table 1).

### 3.3. Association between Iron Intake, Dietary Factors, and Sociodemographic Characteristics

Pearson chi-square showed statistically insignificant (*p* > 0.05) relationship between dietary iron intake and factors such as age (*X*^2^ = 0.584), participants' level of education (*X*^2^ = 0.919), type of meal skipped (*X*^2^ = 0.306), breakfast intake (*X*^2^ = 0.627), and snacking (*X*^2^ = 0.843). Pearson chi-square analysis showed that guardian educational status (*X*^2^ = 6.7,*p* < 0.05), number of times of eating a day (*X*^2^ = 12.6, *p* < 0.05), and meal skipping (*X*^2^ = 5.7, *p* < 0.05) had statistically significant relationship with the dietary iron intake (Table 1).

### 3.4. Principal Components of Iron Intake Patterns

Kaiser–Meyer–Olkin (0.53) and Bartlett's test of sphericity (*p* < 0.05) showed that the data was sufficient for factor analysis. Three factors (patterns) were retained from Cattell's scree plot. The loading factor of each dietary pattern constituent is shown in Table 2. The three patterns identified were named ‘iron dietary pattern one', ‘iron dietary pattern two,' and ‘iron dietary pattern three'; unitedly, they explained 28.7% of the dietary iron intake variance (Table 2). Iron dietary pattern (DP) one (iron DP 1) explained 11.7% of the variance and was characterised by eggs, cocoyam leaves, turkey berries, dandelion, carrot, tangerine, pineapple, pawpaw, guava, pepper, tomato, coffee, soft drinks, sugar-sweetened beverages, black tea, and chocolate. Iron dietary pattern two (iron DP 2) explained 9.9% of the variance and comprised high consumption of meat, fish, poultry, egg, *Amaranthus* leaves, dandelion, moringa, oranges, lemon, with low intake of soft drinks, sugar-sweetened beverages, black tea, and chocolate. Iron dietary pattern three (iron DP 3) explained 7.1% of the variance and was composed of high intake of stinging nettle, coffee, soft drinks, sugar-sweetened beverages, with low intake of cocoyam leaves, turkey berries, pepper, and tomato.

Factor analysis (extraction method: principal component analysis; rotation method: varimax with Kaiser normalisation). Loading factor: ±0.3. DP 1: varying foods such as iron-rich, iron-enhancing, and iron-inhibiting foods; DP 2: iron-rich, iron-enhancing, and non-iron-inhibiting foods; DP 3: stinging nettle, iron-inhibiting foods, non-iron-enhancing foods, non-cocoyam leaves, and non-turkey berries.

### 3.5. Iron Status

Table 3 shows biochemical markers and dietary iron intake. Mann–Whitney U test showed statistically significant (*p* < 0.05) relationship at haemoglobin level and ferritin level. Haemoglobin (Hb), ferritin, and C-reactive protein had median values of 11.8 (5.1–14.5), 43.0 (2.8–272.0), and 3,091.0 (1,030.0–7,420.0), respectively. Pearson chi-square test showed no statistically significant (*p* > 0.05) relationship in haemoglobin status and ferritin status. The study revealed that 70.6% of the adolescents had normal Hb and 29.4% had anaemia. Among the normal Hb group, 63.5% had adequate iron intake and 36.5% had inadequate dietary iron intake. Approximately 98% of the participants had normal ferritin. In the normal ferritin group, 36.7% had adequate dietary iron intake and 63.3% had inadequate intake. C-reactive protein levels of participants were normal with 62.6% having adequate dietary iron intake and 37.4% having inadequate intake.

### 3.6. Association between Patterns of Iron Intake and Serum Iron Status

After being adjusted for age and gender, partial correlation showed weak inverse and statistically significant relationship between dietary iron intake and iron DP 1 (*r* = −0.234*∗∗*, *p* < 0.01), dietary iron intake and iron DP 2 (*r* = -0.198*∗*, *p* < 0.05), and iron DP 2 and vitamin C intake (*r* = −0.201*∗*, *p* < 0.05). Positive partial correlation and statistically significant relationship existed between dietary iron intake and dietary vitamin C intake (*r* = 0.520*∗∗*, *p* < 0.01). There was weak negative partial correlation between dietary iron intake and ferritin (*r* = −0.124), and dietary iron intake and C-reactive protein (*r* = −0.013) (Table 4).

Haemoglobin levels revealed weak and negative partial correlation, respectively, with iron DP 1 (*r* = −0.075), iron DP 2 (*r* = −0.086), and iron DP 3 (*r* = −0.093). Haemoglobin levels showed weak positive partial relationship between dietary iron intake (*r* = 0.084) and vitamin C (*r* = 0.090), respectively. Ferritin and haemoglobin showed statistically significant and weak inverse correlation (*r* = −0.200*∗*, *p* < 0.05) (Table 4).

### 3.7. Predictors of Iron Status

Binary logistics regression showed that sociodemographic and dietary factors had insignificant relationship (*p* > 0.05) with haemoglobin status (Table 5). The odds of anaemia were nonsignificantly lower among male participants (OR = 0.6, *p*=0.193, 95% CI = 0.3–1.3), guardians with formal education (OR = 0.4, *p*=0.075, 95% CI = 0.1–1.1), breakfast takers (OR = 0.9, *p*=0.864, 95% CI = 0.3–2.6), and participants with adequate vitamin C intake (OR = 0.9, *p*=0.926, 95% CI = 0.2–5.0). Furthermore, the odds of anaemia were nonsignificantly higher among JHS participants (OR = 1.7, *p*=0.213, 95% CI = 0.7–4.0), meal skippers (OR = 1.4, *p*=0.328, 95% CI = 0.7–3.0), and adolescents who snacks (OR = 1.6, *p*=0.234, 95% CI = 0.7–3.6).

## 4. Discussion

The study identified patterns of dietary iron intake, iron status, and predictors of anaemia among early adolescents in a rural district in Ghana.

The application of principal component analysis (PCA) in the study identified three iron dietary patterns, namely, iron dietary pattern one, iron dietary pattern two, and iron dietary pattern three. All of the three iron dietary patterns were inversely related to haemoglobin levels and dietary iron intake. However, there was a significant relationship between dietary iron intake and iron dietary patterns one and two, respectively. Guardians with formal education, the number of times of eating a day, and meal skipping were significantly associated with dietary iron intake. Dietary iron intake was significantly related to dietary vitamin C intake. Male gender, a guardian with formal education, breakfast, and dietary vitamin C intake reduced the rate of anaemia occurrence; however, participants with JHS education, meal skipping, and snacking habit showed the contrary. All the sociodemographic and dietary factors showed no statistically significant relationship with the rate of anaemia.

Iron dietary pattern one was characterised by eggs, cocoyam leaves, turkey berries, dandelion, carrot, tangerine, pineapple, pawpaw, guava, pepper, tomato, coffee, soft drinks, sugar-sweetened beverages, coffee, black tea, and chocolate. Foods that constituted iron dietary pattern two were meat, fish, poultry, egg, *Amaranthus* leaves, dandelion, moringa, and less or no consumption of soft drinks, sugar-sweetened beverages, black tea, and chocolate. Iron dietary pattern three comprised stinging nettle, coffee, soft drinks, sugar-sweetened beverages, and low intake of cocoyam leaves, turkey berries, pepper, and tomato. Participants with iron dietary pattern two consumed more of the haem iron sources compared to the other patterns. Iron dietary pattern one participants consumed more of the non-haem sources, iron-enhancing foods, and iron-inhibiting foods compared to iron dietary pattern two and three participants. Findings from iron dietary pattern one is consistent with other studies [38, 39]. Frequent intake of iron-inhibiting foods in the iron dietary pattern one had a semblance of “western” and “unhealthy” dietary pattern identified in adolescents in Brazil and Republic of Ireland, respectively [40, 41]. Excessive consumption of non-haem iron food sources as the major sources of iron intake of dietary pattern one and two confirms most of the dietary intake studies in developing countries [15, 42, 43]. The inverse relationship between all the iron dietary patterns and haemoglobin and dietary iron intake may be related to limited consumption of iron-enhancing foods, high consumption of iron-inhibiting foods and non-haem iron sources, and weakness of the dietary survey method as it relies on memory and estimation. Significant relationship and weak inverse correlation between dietary iron intake and iron dietary patterns one and two are evidenced in increased similar intake of iron-rich foods and iron-enhancing foods in these patterns.

Dietary iron intake was statistically significant and positively associated with guardians with formal education and meal skipping. Most guardians are key kitchen persons, and since they could read and understand food labels, this might have encouraged the intake of iron-rich foods and iron-enhancing foods as shown in iron DP 1 and iron DP 2. A similar outcome has been shown in other studies [44, 45]. However, a study reported that adolescents with guardians of less education had adequate dietary iron intake [46]. Meal skipping significantly reduced adequate dietary iron intake, which is consistent with other studies that found that meal skipping significantly reduced intake of iron, folate, calcium, sodium, and fibre [47, 48]. On the contrary, higher vitamin C intake was identified in a disordered eating pattern among teenagers [49].

In this study, the majority of participants had adequate iron intake, and male gender had adequate dietary iron intake, thus showing a positive association with low anaemia prevalence, which has also been reported in other studies [4, 50–53]. Low rate of anaemia in participants with guardians of formal education confirms the role of education in alleviating anaemia [54–57]. The educated guardians could read and understand, hence influencing their children to make healthy and informed choices of food. The present study concurred with other findings that intake of breakfast increased the possibility of normal haemoglobin levels [34, 58]. A study revealed that ‘traditional breakfast' intake significantly influenced micronutrients intake [59]. Adequate vitamin C intake may have helped boost immunity, reduced infections, and enhanced iron absorption, being thus positively associated with high haemoglobin levels. Findings about this effect have been reported in a scientific study [60]. The present study reveals that inadequate dietary iron intake by females, in addition to menarche and menstrual blood loss, might have resulted in low haemoglobin levels [31]. Inadequate dietary iron intake was also observed among European adolescent girls [61]. The current study adds to several others that revealed high levels of anaemia in female participants due to similar factors [62–64].

Meal skipping was popular among our study population as seen in most studies [65–68]. Breakfast and dinner were skipped; however, lunch was the most skipped meal as has been reported in other scientific studies [67, 69]. The present study shows that meal skipping was related to an inadequate iron intake which concurs with previous studies that showed that meal skipping contributed to poor micronutrient intake in adolescents [33, 56, 66]. The current study shows that meal skipping was highly associated with anaemia due to inadequate intake of iron-rich and iron-enhancing foods as seen in iron DP 3. Participants at JHS level showing a high rate of anaemia may be due to meal skipping, having guardians with nonformal education, and excessive consumption of iron-inhibiting foods exhibited in iron dietary patterns one and three. This finding contradicts the outcome of other studies [70, 71]. Increased snacking showed high inadequate dietary iron intake, and it was consistent with high rate of low haemoglobin levels as most participants reported consuming soft drinks, popcorn, cocoa tea, biscuits, toffees, ice cream, and plantain chips. Similar choices of snacking were found in adolescents living in other developing countries [72, 73]. Snacking is a recipe for poor dieting in adolescent [74]. It was also evident in the iron dietary pattern one as most of the participants consumed all the varieties of iron-inhibiting foods.

All the participants had normal C-reactive protein levels with the majority having normal ferritin and haemoglobin levels. C-reactive protein showed a weak inverse relationship with haemoglobin. Dietary iron and vitamin C consumption had a positive moderate correlation and statistically significant relationship. Our result shows that participants with dietary iron and vitamin C intake had a weak positive correlation with haemoglobin levels, confirming other scientific findings [75, 76]. Moreover, these factors had a negative association with anaemia. It may be possible that most of these participants belong to iron dietary pattern two as high consumption of haem iron and iron-enhancing food was seen in this pattern. The study outcome appears to support the recommendation of encouragement of dietary iron intake and vitamin C to enhance haemoglobin levels [77]. Ferritin showed a weak inverse correlation with dietary iron and vitamin C intake. This may be due to inadequate intake of foods rich in iron and vitamin C, as seen in iron DP 3, and high consumption of iron-inhibiting and non-haem iron foods, as demonstrated in iron DP 1. Plant sources of iron have low bioavailability, whereas haem iron is shown to be a better predictor of serum ferritin in an Australian population study [78, 79].

## 5. Conclusion

The iron dietary patterns showed a high intake of non-haem iron and iron-inhibiting foods. Meal skipping, guardians with formal education, and times of eating a day were significantly related to dietary iron intake. Meal skipping, snacking, and adolescents with JHS education were more associated with low haemoglobin levels.

## Figures and Tables

**Table 1:** Sociodemographic factors, dietary factors, and dietary iron intake.

Total variables	Mean ± SD	*N* (%)	Dietary iron intake			*X* ^2^	*p* value
			Adequate		Inadequate		
Age (years)	11.5 ± 1.1					1.9	0.584
10		30 (21.9)	19 (22.2)		11 (21.6)		
11		42 (30.7)	23 (26.7)		19 (37.3)		
12		32 (23.3)	21 (24.4)		11 (21.6)		
13		33 (24.1)	23 (26.7)		10 (19.5)		

Level of education							
Participants						0.0	0.919
Primary		96 (70.1)	60 (69.8)		36 (70.6)		
Junior high school		41 (29.9)	26 (30.2)		15 (29.4)		

Guardian						6.7	**0.010**
Formal education		106 (77.9)	61 (70.9)		45 (90.0)		
Nonformal education		30 (22.1)	25 (29.1)		5 (10.0)		

Guardian						3.5	0.319
Both parents		88 (64.2)	59 (68.6)		29 (56.9)		
Mother		24 (17.5)	12 (14.0)		12 (23.5)		
Father		5 (3.7)	4 (4.6)		1 (2.0)		
*∗* Others		20 (14.6)	11 (12.8)		9 (17.6)		

Times of eating a day						12.6	**0.002**
Two		19 (13.9)	8 (9.3)		11 (21.6)		
Three		98 (71.5)	59 (68.6)		39 (78.0)		
Four or more		20 (14.6)	19 (22.1)		1 (0.4)		

Meal skipping						5.7	**0.021**
Yes		76 (55.5)	41 (47.7)		35 (68.6)		
No		61 (44.5)	45 (52.3)		16 (31.4)		

Type of meal skipped						2.4	0.306
Breakfast		15 (19.7)	10 (24.4)		5 (14.3)		
Lunch		57 (75.0)	30 (73.2)		27 (77.1)		
Dinner		4 (5.3)	1 (2.4)		3 (8.6)		
Total		76 (100)	41 (53.9)		35 (46.1)		

Breakfast today						0.3	0.627
Yes		116 (84.7)	74 (86.0)		42 (82.4)		
No		21 (15.3)	12 (14.0)		9 (17.6)		
Total		137 (100)	86 (62.8)		51 (37.2)		

Snacking						0.1	0.843
Yes		100 (73.0)	62 (72.1)		38 (74.5)		
No		37 (27.0)	24 (27.9)		13 (25.5)		
Total		137 (100)	86 (62.8)		51 (37.2)		

*∗*Others: grandparent, aunt, uncle. Data are presented as frequency (percentage), mean, and standard deviation (SD). Pearson chi-square value is significant at *p* < 0.05.

**Table 2:** Principal component analysis of dietary pattern (DP) of iron-rich food consumed.

Food groups	DP1	DP2	DP3
*Iron-rich*			
Meat		0.599	
Fish		0.665	
Poultry		0.373	
Egg	0.344	0.349	
Cocoyam leaves	0.321		−0.459
Turkey berries	0.403		−0.497
Amaranthus leaves		0.408	
Stinging nettle			0.332
Dandelion	0.408	0.358	
Moringa		0.428	
Carrot	0.318		

*Iron-Enhancing*			
Oranges		0.345	
Lemon		0.381	
Tangerine	0.36		
Pineapple	0.468		
Pawpaw	0.356		
Guava	0.451		
Pepper	0.367		0.349
Tomato	0.488		0.334

*Iron-inhibiting*			
Coffee	0.47		0.378
Soft drinks	0.469	−0.305	0.567
Sugar-sweetened beverages	0.51	−0.433	0.433
Black tea	0.343	−0.438	
Chocolate	0.48	−0.415	
% variance	**11.70%**	**9.90%**	**7.10%**
% accumulated variance	**11.70%**	**21.60%**	**28.70%**

**Table 3:** Biochemical markers and dietary iron intake.

Total variables	Median (min–max)	*p* value	*N* (%)	Dietary iron intake		*X* ^2^	*p* value
				Adequate	Inadequate		
Haemoglobin	11.8 (5.1–14.5)	<**0.05**				0.0	1.000
No anaemia			96 (70.6)	61 (63.5)	35 (36.5)		
Anaemia			40 (29.4)	25 (62.5)	15 (37.5)		

Ferritin	43.0 (2.8–272.0)	<**0.05**				1.1	0.556
Low			3 (2.3)	1 (66.7)	2 (33.3)		
Normal			128 (97.7)	47 (36.7)	81 (63.3)		

C-reactive protein	3091.0 (1030.0–7420.0)						
Normal			131 (100)	82 (62.6)	49 (37.4)		

Continuous nonparametric data are presented in median (minimum–maximum); Mann–Whitney U test; chi-square test; frequency (percentage); Fisher's exact. *p* value is significant at *p* < 0.05.

**Table 4:** Partial correlation of iron dietary pattern (IDP) and biochemical markers.

Variables	Iron			Dietary iron	Vitamin C	Haemoglobin	Ferritin	C-protein
	DP 1 reactive	DP 2	DP 3					
Iron DP 1	1.000	−0.047	0.045	−0.234^*∗∗*^	−0.080	−0.075	0.099	−0.005
Iron DP 2	−0.047	1.000	0.030	−0.198^*∗*^	−0.201^*∗*^	−0.086	−0.075	−0.081
Iron DP 3	0.045	0.030	1.000	−0.080	−0.087	−0.093	0.014	0.055
Dietary iron	−0.234^*∗∗*^	0.198^*∗*^	−0.080	1.000	0.520^*∗∗*^	0.084	−0.123	0.010
Vitamin C	−0.080	−0.201	−0.087	0.520^*∗∗*^	1.000	0.090	−0.015	0.053
Haemoglobin	−0.075	−0.086	−0.093	0.084	0.090	1.000	−0.178^*∗*^	−0.140
Ferritin	0.099	−0.075	0.014	−0.123	−0.015	−0.178^*∗*^	1.000	0.100
C-reactive protein	−0.005	−0.081	0.055	0.010	0.053	−0.140	0.100	1.000

Controlled variables: age and gender. ^*∗*^Partial correlation is significant at the 0.05 level (2-tailed). ^*∗∗*^Partial correlation is significant at the 0.01 level (2-tailed). DP: dietary pattern.

**Table 5:** Binary logistic regression for predictors of anaemia.

Predictors	*β*	Anaemia OR (95% CI)	*p* value
*SociodemographicsGender*			
Male	−0.492	0.6 (0.3–1.3)	0.193
Female		1	

*Level of education*			
JHS	0.544	1.7 (0.7–4.0)	0.213
Primary		1	

*Guardian educational status*			
Formal	−0.944	0.4 (0.1–1.1)	0.075
Nonformal education		1	

*Dietary factors*			
Meal skipping			
Yes	0.367	1.4 (0.7–3.0)	0.328
No	1		

*Breakfast*			
Yes	−0.090	0.9 (0.3–2.6)	0.864
No	1		

*Snacking*			
Yes	0.484	1.6 (0.7–3.6)	0.234
No	1		

*Dietary iron intake*			
Adequate	−0.011	1.0 (0.5–2.1)	0.977
Inadequate	1		

*Dietary vitamin C intake*			
Adequate	−0.080	0.9 (0.2–5.0)	0.926
Inadequate	1		

*p* < 0.05; 95% CI: confidence interval; OR: odd ratio.

## Data Availability

The data used to support the findings of this study could be assessed by writing to the Chairman Committee on Human Research Publication Ethics, Room 8 Anatomy Block 3, School of Medical Sciences, Kwame Nkrumah University of Science and Technology, Kumasi, Ghana, or chrpe.knust.kath@gmail.com.
